# Liposomal Circular Dichroism (L-CD) of Arenoyl Derivatives of Sphingolipids. Amplification of Cotton Effects in Ordered Lipid Bilayers

**DOI:** 10.3390/md15120352

**Published:** 2017-12-20

**Authors:** Tadeusz F. Molinski, Caroline D. Broaddus, Brandon I. Morinaka

**Affiliations:** 1 Department of Chemistry and Biochemistry, University of California, San Diego, 9500 Gilman Drive MC0358, La Jolla, CA 92093, USA; broaddusc@yahoo.com (C.D.B.); phambi@nus.edu.sg (B.I.M.); 2 Skaggs School of Pharmacy and Pharmaceutical Sciences, University of California, San Diego, 9500 Gilman Drive MC0358, La Jolla, CA 92093, USA

**Keywords:** marine natural product, circular dichroism, exciton coupling, absolute configuration, liposome, sphingosine, sphingolipid

## Abstract

Liposomal circular dichroism (L-CD) of acyclic amino alcohols exhibit amplification of Cotton effects when measured in highly uniform, unilamellar liposomes. The effect is likely due to intermolecular associations—H-aggregates—that self-assemble spontaneously within the lipid bilayer, and persists over long time scales. L-CD spectra of *N*,*O*,*O*′-tri-(6′methoxy-2′-naphthoyl)-d-*erythro*-sphingosine, or the corresponding dihydro-derivative (sphinganine), shows ~10-fold amplification of magnitudes of Cotton effects over conventional CD spectra recorded in isotropic solution.

## 1. Introduction

**Figure 1:**
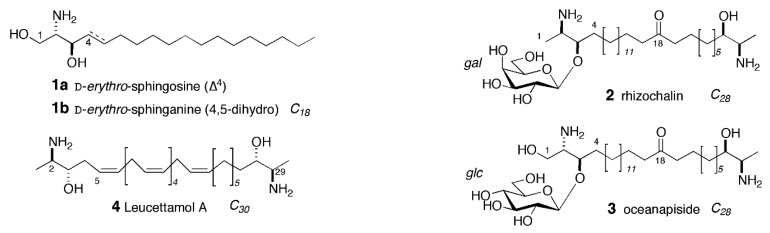
d-Sphingosine (**1a**), its dihydro-derivative, sphinganine (**1b**), and “two-headed” sphingolipid natural products.

The assignment of relative configuration (RC) and absolute configuration (AC) of marine natural products, in particular long-chain flexible polyketides and lipids, has no general solution; stereochemical elucidation is approached on a case-by-case basis. Creative integrated approaches, based on a number of methods (e.g., modified Mosher’s ester method for secondary alkanols [1,2], degradation-synthesis, nOe and J-based NMR approaches [3], circular dichroism [4,5] (CD)—and more recently, vibrational circular dichroism (VCD) [6]—have often led to successful outcomes in numerous instances [7]. CD, with comparisons of synthetic model compounds of known configuration, has been used to assign AC in long-chain lipids with native chromophores, albeit with weak Cotton effects (CEs) [8]. Interpretation of exciton coupled circular dichroism (ECCD) of perbenzoates of acyclic polyols [9,10,11], and benzoate/benzamide pairs of vicinal aminoalkanols [12] give rise to characteristic bisignate Cotton effects (CEs) that reflect their absolute configuration [13]. These methods, largely developed by Nakanishi, Berova, and Gawronski, exploited pair-wise intramolecular exciton coupling to develop “fingerprint” CD spectra of all possible diastereomeric combinations of polyols, and 2-amino-3-alkanols and 2-amino-1,3-alkanediols—sphingolipids—and has been applied to the assignment of absolute configuration of d-*erythro*-sphingosine (**1a**, Figure 1), sphinganine (**1b**) and related acylic natural products [14,15,16].

More complex C_28_ and C_30_ sphingolipids—the so-called antifungal “two-headed” sphingolipids [17,18,19,20]—which comprise pseudo-dimeric α,ω-bis-aminoalkanols with four or more stereocenters of heterogeneous configurations—have also been addressed by empirical variants of this method. We showed that deconvolution of complex superposed CEs (a corollary of van’t Hoff’s principle of optical superposition) [21] enables assignment of absolute and relative configurations of four stereocenters in two-headed sphingolipids by matching their measured spectra against calculated “hybrid-CD spectra” generated by linear combinations of simple *threo* and *erythro* benzoyl derivatives of diastereomeric 2-amino-3-alkanol and 2-amino-1,3-alkanediol models [20,21].

The latter method has been applied to the complete stereo-assignments of calyxoside from the red alga, *Calyx* sp. [18], and the sponge natural products rhizochalin (**2**) and rhizochalinins [20,21] from *Rhizochalina incrustata* [22], oceanapiside (**3**) from *Oceanapia phillipensis* [23,24], leucettamol A (**4**) from *Leucetta microrhaphis* [25], and the truncated-chain sphingolipid, (2*S*,3*R*)-2-aminodocecan-3-ol from the tunicate, *Clavelina oblonga* [26]. In the latter examples, the CEs arise from pair-wise intramolecular exciton coupling of benzoate/benzamide chromophores and their magnitudes are modest to weak, up to ∆ε~7.5–11 mol^−1^·dm^3^·cm^−1^ for the perbenzoyl d-*erythro*-sphingosine (the *threo* isomer is weaker). The corresponding 1,3-dinaphthoate/2-naphthamide pairs show larger dichroism, with ∆ε values up to 56–68.6 mol^−1^·dm^3^·cm^−1^, or 5–9-fold higher in magnitude for a comparable model compound of the same configuration [27].

Although the above CD methods for acyclic systems are sensitive (the sample requirement is of the order of µg) the critical drawback is still the limit of detection. Free bond rotation and other degrees of freedom can average out CEs that arise from weak perturbations of chromophores within an asymmetric sphere, or even the stronger pair-wise exciton couplings, although—as we have shown above—this can be partially compensated for by use of chromophores with stronger electronic transition dipole moments [12]. Here, we describe a CD method that produced significant gains in the magnitudes of the Cotton effects of *N*,*O*,*O*′-triacyl sphingolipids when their CD spectra were measured in liposomal formulations. When arrayed in uniform, unilamellar liposomes, prepared by membrane extrusion methods, the membrane-bound lipids, bearing arylcarboxylate chromophores, assemble into ordered arrays that exhibit intermolecular interactions through delocalized excitons—the so-called J- and H-aggregates [28,29]. The corresponding liposomal circular dichroism (L-CD) spectra exhibit more complex features including longer wavelength CEs with dramatic amplifications of magnitude.

## 2. Results

**Scheme 1:**
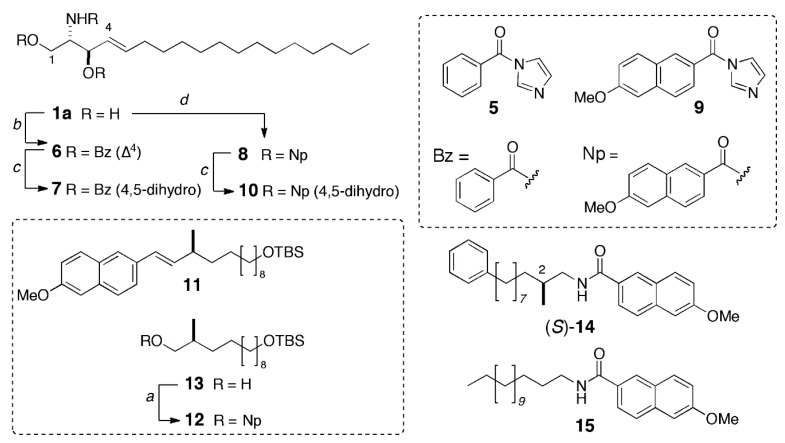
Synthesis of sphingolipid benzoyl and (6-methoxy-2-naphthoyl) derivatives. *a*, EDCI, DMAP, 6-methoxy-2-naphthoic acid, CH_2_Cl_2_; *b*, *N*-benzoylimidazole (**5**), DBU, CH_2_Cl_2_; *c*, H_2_ (1 atm), 10% Pd-C, EtOAc; *d*, *N*-(6′-methoxy-2′-naphthoyl)imidazole (**9**), DBU, CH_2_Cl_2_.

For the purposes of this study, model compounds containing benzoyl or 6-methoxy-2-naphthoyl chromophores (herein, abbreviated Np) were prepared (Scheme 1) for measurements of CD spectra under two sets of conditions: isotropic media (MeOH) or liposomal formulations (L-CD, liposomes 18:0), as previously described [30], where the DPPC or DSPC [31] bilayers comprise an anisotropic medium [32,33]. Natural d-sphingosine (**1a**) was converted (*N*-benzoylimidazole (**5**), DBU, CH_3_CN, 70 °C) to the corresponding perbenzoate (**6**) which was subsequently hydrogenated (H_2_, 1 atm, 10% Pd-C, EtOAc, 23 °C) to give *N*,*O*,*O*′-tribenzoylsphinganine (**7**) [16]. A parallel sequence of reactions gave pernaphthoyl derivative, **8**, by acylation of **1a** (*N*-Np-imidazole, **9**, DBU, CH_3_CN, 60 °C), which was subsequently hydrogenated under the above conditions to give the pernaphthoyl sphinganine **10**. For purposes of comparison, the long-chain 2-vinylnaphthalene **11** and the simple primary Np ester **12**, prepared from the known long-chain primary alcohol **13** [7], were also secured.

The CD spectra of the **6**–**8**, **10**–**12** were measured under two sets of conditions: isotropic (MeOH solution) and anisotropic conditions obtained by formulation into membrane-bound unilamellar liposomes. Each of the latter samples was prepared from crude liposomes (sonication) by repeated extrusion through a porous membrane (100 Å pore size), which, as shown previously [30,32,33], gave uniform spherical liposomes of approximately 25–30 nm in diameter. Measurement of chiroptical properties of these uniform nanoparticles can be made with little or no light scattering, and reveal complex features (absent from CD spectra recorded under isotropic conditions) that we attribute to intermolecular excitons.

**Figure 2:**
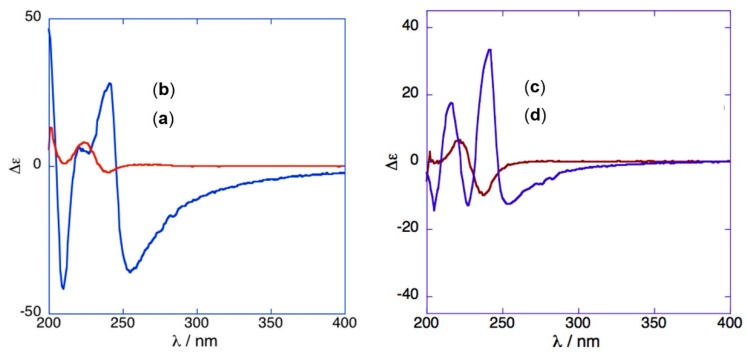
Circular dichroism (CD) spectra (23 °C) of (a) (red) *N*,*O*,*O*′-tri-(benzoyl)-d-sphingosine (**6**) in MeOH (*c* = 6.17 × 10^−5^ M) and (b) (blue) **6** in DSPC liposomes (*c* = 1.63 × 10^−4^ M) (c) (red) tri-(benzoyl)-d-sphinganine (**7**) in MeOH (*c* = 2.2 × 10^−4^ M) and (d) (blue) **7** in DSPC liposomes (*c* = 5.28 × 10^−5^ M).

The CD spectrum of *N*,*O*,*O*′-tribenzoyl sphingosine **6** in MeOH (Figure 2a, Table 1) has relatively simple features: bisignate peaks due to a simple ECCD effect of relatively weak magnitude (∆ε +8.2 (224 nm), −2.2 (240 nm)), and a short-wavelength edge (λ ~ 205 nm) of a CE masked by end-absorption of the MeOH solvent: essentially identical to that reported from earlier studies [16,23]. In sharp contrast, the L-CD spectrum of the **6**, formulated in DSPC [31] unilamellar liposomes (aqueous), shows more complex behavior dominated by three strong CEs with greatly amplified magnitudes: a shorter wavelength negative band (∆ε −42 (λ = 210 nm), a significantly longer wavelength (∆ε +28.0 (241 nm)) and a strong, negative broad band (λ 255 nm, ∆ε −36 that tails off toward longer wavelengths, and stronger edge features at λ ~ 200 nm compared with **6** in MeOH.

Hydrogenation of the ∆^4^ double bond in **6** to give the tri-benzoylsphinganine derivative **7** gives rise to a CD (MeOH) spectrum (Figure 2c, Table 1) that is qualitatively similar to the latter (Figure 2a)—a simple bisignate CE, but stronger in magnitude (∆ε +6.3 (220 nm), −10.1 (238)). Reconstituting **8** in DSPC [31] liposomes gives rise to a dramatically different L-CD spectrum with complex features dominated by five CEs (∆ε −14 (204 nm), +18 (216), −13.0 (227), +33.3 (242), −12.6 (253)) and smaller edge effects. The latter two CEs occurred at longer wavelengths than those arising from chromophores in isotropic media, and the last one tapered off toward longer wavelengths, as did **6** (Figure 2d).

**Figure 3:**
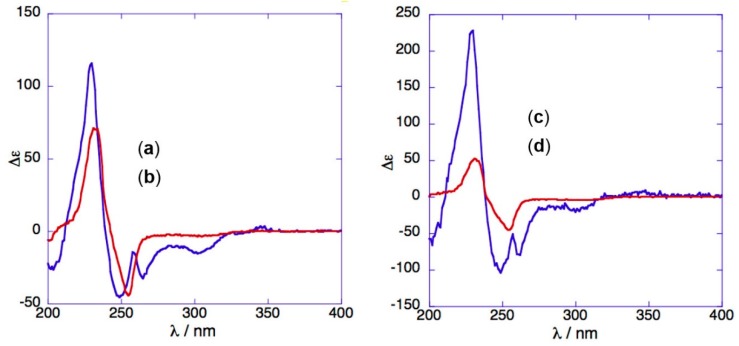
CD spectra (23 °C) of (a) (red) *N*,*O*,*O*′-tri-(6′-methoxy-2′-naphthoyl)-d-sphingosine (**8**) in MeOH (*c* = 1.4 × 10^−5^ M) and (b) (blue) **8** in DSPC liposomes (*c* = 5.87 × 10^−5^ M); (c) (red) *tris*-(6′-methoxy-2′-naphthoyl)-d-sphinganine (**10**) in MeOH (*c* = 1.4 × 10^−5^ M) and (d) (blue) **10** in DSPC liposomes (*c* = 1.4 × 10^−5^ M).

Replacement of the Bz groups in **6** and **7** with Np groups–the triacyl sphingosine **8** and sphinganine **10**—gave isotropic CD spectra in MeOH (Figure 3a,c, respectively, Table 1) qualitatively similar to **6** and **7** (e.g., **10**: ∆ε +52 (231 nm), −46 (255)), but with CEs of higher magnitudes. Under L-CD conditions, the CD spectra of **8** and **10** (Figure 3b,d, respectively)—to our surprise—were not even similar to the L-CD spectra of tri-benzoyl derivatives **6** and **7**. In the L-CD spectra of **8** and **10**, the long-wavelength features were diminished and, in both compounds, the CEs collapsed into a single dominant, more intense broad band; for example, in **10** (∆ε +227 (230 nm)), close to the positive CE of **6** and **7**, along with two less intense negative CEs (∆ε −104 (249), −80 (262)).

Finally, measurement of the CD spectra of **11** and **12** (Table 1), either in MeOH or under L-CD conditions (DSPC), showed no CEs, only baseline. Reformulation of **11** into liposomes, prepared from fatty acyl phosphatidylglycerols of differing chain lengths (DLPC, DMPC, DPPC, DSPC) [31], did not change the spectra. On occasion, we have observed that the ordered self-assembly of long-chains appended with arylcarboxylate chromophores in liposomes [32] is kinetically limited and requires annealing over time or temperature, however, in the case of **11** and **12**, CEs did not appear in the respective L-CD spectra, even after 24 h at 23 °C.

## 3. Discussion

CD spectra of perbenzoyl sphingolipids **6** and **7** in MeOH solution (Table 1) show the expected bisignate split-Cotton effects. The new derivatives **8** and **10**, bearing the Np chromophore bearing donor-acceptor substituents, confer higher oscillator strengths for the intramolecular charge transfer transition (the ^1^*B*_b_ band) than most *p*-substituted benzoates or 2′-naphthoates. Consequently, the interactions between Np ester-ester and ester-amide pairs show superposed CEs of higher absolute magnitudes than the corresponding Bz derivatives with more pronounced, symmetric bisignate split CEs. For example, the absolute magnitude of peak-to-trough ∆ε values (the *A* parameter) for **8** and **10** (Entries 3 and 4) are 114 and 97.4, respectively, compared to only 10.4 and 16.4 for **6** and **7**, (Entries 1 and 2), respectively. Given the ~10-fold greater magnitude of CEs, and comparable ease of Np derivatization and per-benzoylation, we recommend Np as a chromophore over Bz or 2-naphthoyl for AC assignments of aminoalkanols using the canonical “dibenzoate method” [13].

**Figure 4:**
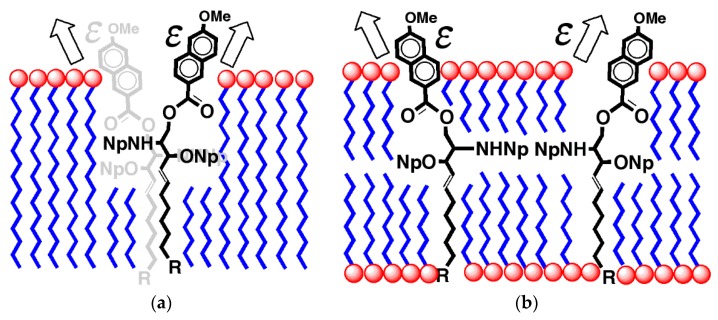
Cartoon of hypothetical intermolecular interactions between paired *N*,*O*,*O*′-tri-acylsphingosine molecules within the liposomal bilayer. Red spheres = polar head groups of DSPC, Np = 6-methoxy-2-naphthoyl, ε = electronic transition dipole moment (charge transfer band). For clarity, only two Np groups are shown, and intramolecular interactions are not depicted. (**a**) Close packed chromophores (**b**) lipid chain-separated chromophores.

All L-CD spectra recorded for sphingosine-sphinganine pairs, **6**, **7** and **8**, **10**, showed dramatically amplified CEs with new bands appearing at longer wavelengths compared to those observed in MeOH. Two factors contribute to these phenomena: restricted rotation of the chromophore-appended head-groups of sphingolipids that impedes conformational averaging, and intermolecular assembly of membrane-bound chromophores into H-aggregates. This assembly of triacyl-sphingolipids, largely driven by dipole-dipole and π-π stacking interactions (Figure 4), leads to delocalized chromophores with longer wavelength absorptions. These assemblies, normally weak in isotropic dilute solution where solvent-solute interactions dominate, are consolidated and strengthened by hydrophobic packing forces (and possibly augmented by hydrogen bonding) that organize the long-chain tails within the interior of the liposomal lipid bilayer. Inherent amplification of chirality through self-organizing polymers or oligomers has been reported before [34], but the use of liposomes in the present context achieves a similar effect and adds another dimension to CD as a tool to augment stereoassignment.

The orientations of the electronic transition dipole moments ε of the arenoyl groups (Figure 4; only the primary *O*-Np groups are shown) within a monomer and between monomers are not known; indeed, this is a complex problem. Nevertheless, first order ensembles (considering only pairs) are likely offset in a manner reminiscent of conjugated monomeric molecules in other H-aggregates [35]. Although the bilayer is considered a “fluid mosaic”, closeness of spacing of paired monomeric chromophores (e.g., close packed form, Figure 4a) should influence the degree of delocalization of excitons. Further refinement of this model, beyond that implied in a simplified pair-wise interaction depicted in Figure 4, is wanting, but beyond the scope of this paper.

Earlier, we showed that appearance of the CEs in L-CD of substituted naphthamide **14** [33] was dependent only upon the configuration at C-2 (*R* and *S* enantiomers showed opposite CD spectra of equal magnitude), and exhibited a reversible temperature dependence that followed the expected gel phase transition temperature of DSPC that comprise the liposomes. It is likely that similar non-bonded interactions are also operative in the liposomal formulations of **6**, **7**, **8** and **10**. It is important to note again that racemic (±)-**14** or achiral naphthamide **15** show no CEs (baseline only) in MeOH or in DSPC liposomes, proving that the asymmetry of the bilayer itself is not the origin of induced CEs in L-CD [33].

The strongest amplification of CEs under L-CD conditions was observed in **10**. We estimated the limit of detection (LOD) from the peak-to-trough value of ∆ε for the major bisignate components in the L-CD spectrum of **10** (Figure 2c, *A* = 334), the average noise level from λ = 300–400 nm and the nominal cuvette fill volume (*l* = 2 mm, *V*_f_ = 0.2 mL). Under L-CD conditions the limit of detection of **10** is estimated to be ~56 pmole, or almost 4-fold lower than **10** in MeOH. It is conceivable that the LOD may be further improved by appropriate replacement of the Np group with chromophores of higher oscillator strength, e.g., 2-anthracenoyl, 5-acetyl-7-dimethylamino-2*H*-chromen-2-one, *p*-methoxycinnamoyl [15].

## 4. Materials and Methods

General methods can be found elsewhere [36]. All CD spectra were recorded on a Jasco J-810 spectropolarimeter at 23 °C. NMR spectra were recorded on a Varian Mercury 400 (^1^H NMR, 400 MHz; ^13^C NMR, 100 MHz) with a dual-tuned ^1^H-^13^C 5 mm room temperature probe, or a Jeol ECA 500 with a 5 mm ^1^H{^13^C} inverse detect probe (^1^H NMR, 500 MHz; ^13^C NMR, 125 MHz). Chemical shifts are referenced to internal solvent peaks (residual CHCl_3_, δ_H_ 7.24 ppm; CDCl_3_, δ_C_ 77.0 ppm).

*(S)(+)-12-(tert-Butyldimethylsilyloxy)-2-methyldodecyl 6-methoxy-2-naphthoate* (**12**). A solution of alcohol **13** (6 mg, 0.0181 mmol), 4-dimethylaminopyridine (0.54 mg, 0.44 µmol) and 6-methoxy-2-naphthoic acid (5 mg, 0.0247 mmol) in CH_2_Cl_2_ (0.50 mL) was cooled to 0 °C and treated with EDCI (1-ethyl-3-(3-dimethylaminopropyl) carbodiimide, 2.7 mg, 1.74 µmol) and the mixture was stirred at room temperature overnight. The solvent was removed under a stream of N_2_, and the residue was separated using preparative thin layer chromatography (1:9 EtOAc/hexanes) to give ester **12** (1.12 mg, 13%). [α]_D_ +1.29 (*c* 1.63, CHCl_3_). ^1^H NMR (CDCl_3_, 500 MHz) δ 8.52 (s, 1H), 8.04 (dd, *J* = 8.6, 1.8 Hz, 1H), 7.85 (d, *J* = 8.6 Hz, 1H), 7.76 (d, *J* = 9.2 Hz, 1H), 7.20 (dd, *J* = 9.2, 2.6 Hz, 1H), 7.16 (d, *J* = 2.6 Hz, 1H), 4.26 (dd, *J* = 10.3, 5.8 Hz, 1H), 4.15 (dd, *J* = 10.3, 6.9 Hz, 1H), 3.59 (t, *J* = 6.9 Hz, 2H), 1.63 (m, 1H), 1.50 (m, 1H), 1.31–1.25 (m, 20 H), 1.05 (d, *J* = 6.8 Hz, 3H), 0.89 (s, 6H), 0.04 (s, 9H).

*N-Benzoylimidazole* (**5**). The method of Nakanishi and coworkers was adapted, for the preparation of **5** [37]. A solution of benzoyl chloride (2 mL) in benzene (20 mL) was added dropwise to a solution of imidazole (2.32 g, 34.1 mmol) and benzene (200 mL). The resulting mixture was cooled to 8 °C, then allowed to warm to room temperature and stirred overnight. After removal of solvent, pure **5** was obtained as a clear oil. The ^1^H NMR data were consistent with literature values [27].

*d-(E)-(–)-erythro-N,O,O′-(Tribenzoyl)sphingosine* (**6**) [16]. A solution of d-*erythro*-sphingosine (**1a**, 10 mg, 0.03 mmol) and *N*-benzoylimidazole (82 mg, 0.476 mmol) in acetonitrile (5 mL) was treated with DBU (49 µL, 0.33 mmol) and the mixture stirred at 70 °C for 24 h, then at room temperature for 24 h. After removal of solvent, the mixture was separated using flash chromatography (1:9 EtOAc: hexane), which yielded perbenzoylated d-*erythro*-sphingosine (**6** [16], 11.9 mg, 58% yield). [α]_D_ −5.90 (*c* 2.06, CHCl_3_). ^1^H NMR (CDCl_3_, 400 MHz) δ 8.02 (m, 4H), 7.74 (m, 2H), 7.58–7.39 (m, 9H), 6.73 (d, *J* = 8.8 Hz, 1H), 5.96 (dt, *J* = 15.5, 7.8 Hz, 1H), 5.80 (t, *J* = 6.5 Hz, 1H), 5.66 (dd, *J* = 15.4, 7.0 Hz, 1H), 4.94 (m, 1H), 4.72 (dd, *J* = 11.7, 6.4 Hz, 1H), 4.60 (dd, *J* = 11.7, 4.3 Hz, 1H), 2.06 (q, *J* = 7.0 Hz, 2H), 1.35–1.21 (m, 18H), 0.88 (t, *J* = 7.0 Hz, 3H).

*d-erythro-N,O,O′-(Tribenzoyl)sphinganine* (**7**). A mixture of **6** (5.9 mg, mmol) and 10% Pd-C (1 mg) in ethyl acetate (0.6 mL) was stirred under an atmosphere of H_2_ at room temperature for 48 h then filtered (syringe filter, 0.45-µm) to give **7**. The ^1^H NMR and CD spectroscopic data were consistent with literature values [23].

*d-(E)-erythro-N,O,O′-Tri-(6′-methoxy-2′-naphthoyl)sphingosine* (**8**). A mixture of d-*erythro*-sphingosine (**1a**, 11.6 mg, 0.039 mmol), DBU (49 µL, 0.33 mmol), and *N*-(6-methoxy-2-naphthoyl)imidazole (**9**, 108 mg, 0.43 mmol) and dry acetonitrile (5 mL) was stirred overnight at room temperature, then stirred at 60 °C for an additional 24 h. The solution was then concentrated under a stream of Ar and solvent traces were removed under high vacuum for 30 min to give a crude product which was separated by flash chromatography (3:7 EtOAc-hexane) followed by HPLC (silica, 10 × 25 mm column, 3:7 EtOAc-hexane, 4 mL·min^−1^) to give **8**. ^1^H NMR (CDCl_3_, 400 MHz) δ 8.51 (s, 1H), 8.48 (s, 1H), 8.22 (s, 1H), 8.02 (d, *J* = 8.6 Hz, 1H), 7.98 (d, *J* = 8.6 Hz, 1H), 7.82–7.66 (m, 6H), 7.18–7.08 (m, 6H), 6.97 (m, 2H), 6.03 (dt, *J* = 15.6, 7.8 Hz, 1H), 5.93 (t, *J* = 5.3 Hz, 1H), 5.76 (dd, *J* = 15.4, 7.1 Hz, 1H), 5.05 (m, 1H), 4.86 (dd, *J* = 11.7, 5.8 Hz, 1H), 4.75 (dd, *J* = 11.7, 4.6 Hz, 1H), 2.09 (q, *J* = 7.0 Hz, 2H), 1.38–1.17 (m, 18H), 0.88 (t, *J* = 7.0 Hz, 3H).

*N-(6′-Methoxy-2′-naphthoyl)imidazole* (**9**). The method of Nakanishi and coworkers was adapted for the preparation of **9** [38]. A solution of 6-methoxy-2-naphthoyl chloride (147 mg, 0.668 mmol) in toluene (1.5 mL) was added to a suspension of imidazole (91.1 mg, 1.34 mmol) in toluene (5 mL) and the mixture stirred at room temperature overnight. The mixture was filtered through Celite, and the filter bed washed with additional toluene. After removal of solvent from the combined filtrates under reduced pressure, a clear glass of *N*-(6-methoxy-2-naphthoyl)imidazole (**9**) was obtained (108.4 mg, 64%).

*d-erythro-N,O,O′-Tri-(6′-methoxy-2′-naphthoyl)sphinganine* (**10**). Naphthoyl derivative **8** (1.7 mg, mmol) was added to a vial containing a suspension of 10% Pd on carbon (0.3 mg) in EtOAc (0.5 mL). The mixture was stirred under H_2_ (1 atm) at room temperature for 48 h, passed through a 0.45-micron syringe filter, and the solvent removed from the clear solution to obtain **10**. [α]_D_ −1.5 (*c* 0.39, CHCl_3_). ^1^H NMR (CDCl_3_, 400 MHz) δ 8.49 (s, 1H), 8.40 (s, 1H), 8.28 (s, 1H), 8.02 (d, *J* = 8.6 Hz, 1H), 7.91–7.58 (m, 8H), 7.39–7.04 (m, 6H), 5.51 (m, H), 4.98 (m, 1H), 4.83 (dd, *J* = 11.7, 5.8 Hz, 1H), 4.72 (dd, *J* = 11.7, 4.6 Hz, 1H), 2.07 (m, 1H), 1.96 (m, 1H), 1.38–1.17 (m, 18H), 0.88 (t, *J* = 7.0 Hz, 3H).

**Lipsomal CD Measurements.** Liposomal formulations of **6**, **7**, **8**, **10**, **11** and **13** were prepared according our published procedure [30,32,33]. Briefly, crude liposomes–prepared by ‘shell evaporation’ of a CHCl_3_ solution containing compound and DSPC or DPPC [31] (Avanti Polar Lipids, Alabaster, AL, USA) in a round bottom flask, followed by addition of distilled H_2_O and sonication, followed by annealing (heated to 60 °C, cooled to 23 °C; repeated twice)—were repeatedly extruded (×25) through polycarbonate membranes (100 nm pore) using gas-tight syringes (Liposofast, Avestin, Toronto, ON, Canada). Final lipid concentration, *c* = 1 mg/mL. Isotropic CD spectra were measured on solutions in MeOH at close to the same nominal concentration as L-CD spectra. All CD measurements were carried out on prepared samples contained in a quartz cuvette (*l* = 2 mm).

## 5. Conclusions

Measurements of circular dichroism of 2′-naphthoyl and benzoyl derivatives of sphingosine in unilamellar DSPC liposomal preparations gave rise to large increases in the magnitudes of the Cotton effects and longer wavelength absorptions, compared to isotropic solution (MeOH). The dramatic effects are consistent with delocalized excitons: intermolecular associations in H-aggregates that self-assemble spontaneously within the lipid bilayer and persist over long time scales. The effect was not observed in the L-CD spectra of two counter examples measured: a long-chain vinyl naphthalene and a 2-naphthoate ester of a long-chain, methyl branched lipid. The liposomal CD method allows extension of detection limits down to sub-nanomole levels for critical chiroptical stereochemical assignments in sphingolipid natural products.

## Figures and Tables

**Table 1:** Isotropic CD (MeOH) and liposomal CD (L-CD). Summary of Parameters [∆ε (λ)].

Entry	Cmpd.	MeOH		L-CD				
1	**6**	+8.2 (224)	−2.2 (240)	−41.5 (210)	+28.0 (241)	−36.0 (255)	-	-
2	**7**	+6.3 (220)	−10.1 (238)	−14 (204)	+17.6 (216)	−13.0 (227)	+33.3 (242)	−12.6 (253)
3	**8**	+71.2 (232)	−43.7 (255)	+114 (228)	−44.5 (249)	−31.5 (266)	−14.5 (303)	-
4	**10**	+52.2 (231)	−45.2 (255)	+227 (230)	−104 (249)	−80 (262)	−17.4 (301)	-
5	**11**	^1^	^1^	^1^	^1^	-	-	-
6	**12**	^1^	^1^	^1^	^1^	-	-	-
7	(*S*)-**14** ^2^	^1^	^1^	−23.8 (213) ^2^	+34.6 (233) ^2^	-	-	-
8	(±)-**14** ^2^	^1^	^1^	^1^	^1^	-	-	-
9	**15** ^2^	^1^	^1^	^1^	^1^	-	-	-

^1^ Baseline, only; ^2^ Reference [33].
